# A systematic review of interventions targeting *Anopheles stephensi*

**DOI:** 10.12688/wellcomeopenres.23480.1

**Published:** 2024-12-16

**Authors:** Patricia Doumbe Belisse, Alison M Reynolds, David Weetman, Anne L Wilson, Martin J Donnelly

**Affiliations:** 1 Department of Vector Biology, Liverpool School of Tropical Medicine, Pembroke Place, Liverpool, L3 5QA, UK

**Keywords:** An. stephensi, vector control tools, malaria, mosquito densities

## Abstract

**Background:**

*Anopheles stephensi*, a malaria mosquito originally from South Asia and the Middle East, has been expanding across both Asia and Africa in recent decades. The invasion of this species into sub-Saharan Africa is of particular concern given its potential to increase malaria burden, especially in urban environments where *An. stephensi* thrives. Whilst surveillance of this vector in Africa has recently increased markedly there is a need to review the existing methods of *An. stephensi* control so that we can stop, rather than simply monitor, its spread in Africa.

**Methods:**

We searched published papers in PubMed using *An. stephensi* and intervention-specific search terms. Forty-five full-text articles were screened for eligibility and all those that reported the use of interventions against *An. stephensi*, and the effect on malaria incidence, malaria prevalence or vector densities were included in the analysis. All data retrieved from the literature were from the native range of *An. stephensi* and from the period 1995 to 2018.

**Results:**

Fourteen studies which met the inclusion criteria were included in the final analysis. The vector control interventions discussed were bio larvicides (n=3), repellents (n=1), Indoor Residual Spraying (n=2), Insecticide Treated Nets (n=3), insecticide-treated materials other than nets (n=3), the combined use of repellents and mosquito nets (n=1), and combination of biolarvicide and fish (n=1). Outcomes of the studies were primarily vector density (n=10) although some reported malaria incidence and/or prevalence (n=4).

**Conclusions:**

Long-lasting insecticidal nets and indoor residual spraying are effective in controlling, *An. stephensi-*transmitted malaria and reducing vector density, with repellents offering a complementary approach, especially in urban areas where this vector thrives. The private sector can help scale up affordable repellent production in Africa. There is a need to address gaps in cost-effectiveness analysis and gather more epidemiological evidence to better assess the impact of malaria control strategies.

## Plain language summary

The malaria mosquito vector *Anopheles stephensi*, originally from South Asia and the Middle East, has recently spread to Africa particularly in sub-Saharan areas, where it could increase the malaria burden in cities. While detection of this mosquito in Africa has improved, new strategies are needed to control its spread, not only monitor its impact.

We reviewed studies published between 1995 and 2018 following inclusion and exclusion criteria. Fourteen studies met the criteria and looked at control methods such as larvicides (3 studies), repellents (1 study), indoor residual spraying (2 studies), mosquito nets (3 studies), insecticide-treated materials other than nets (3 studies) and combinations of some of these interventions (2 studies). Most of the studies focused on reducing mosquito populations and a few looked at the impact on malaria cases.

Insecticide-treated nets and indoor spraying were shown to be effective against *An. stephensi* malaria transmission. Repellents could also help, particularly in urban areas where the mosquito thrives. The private sector could support access to affordable repellents in Africa. More research is needed to understand how effective and affordable these malaria control tools could be within communities in Africa.

## Introduction

By 2050, it is estimated that 70% of the world’s population will live in urban environments ^1^. In sub-Saharan Africa urban population growth is frequently associated with poor quality housing and inadequate drainage which may result in the proliferation of mosquito breeding sites and subsequently increased malaria cases ^2, 3^. Approximately 45% of the African population are now living in urban settings ^4^, and therefore the recent arrival of the urban-adapted Asian vector *Anopheles stephensi* is an acute concern ^5^. This vector’s ability to thrive in urban settings and breed in man-made containers all year round ^6^ could undermine efforts to control malaria.

*Anopheles stephensi,* formerly confined to South Asia and the Middle East was observed in the Horn of Africa in 2012 ^7^ and in Sri Lanka in 2017 ^8^. More recently, the mosquito was found in Nigeria (2020), Kenya (2022) and Ghana (2022) ^5, 9– 11^. As an efficient vector of both *Plasmodium falciparum* and *P. vivax*, *An. stephensi* sustains malaria transmission in most of its native range in the Middle East ^12– 15^, India ^16, 17^, and Pakistan ^18^. The potential role of *An. stephensi* in the transmission of malaria in Africa was reported in Djibouti where it is now thought to be responsible for sustained annual transmission ^7, 19^ and then subsequently in Ethiopia ^20, 21^.

The core interventions against *An. stephensi* in its native range are Insecticide Treated nets (ITNs) and Indoor Residual Spraying (IRS) ^22^. Unfortunately, extensive resistance of *An. stephensi* to different insecticides has been reported ^23^, including to DDT, malathion, pyrethroid and carbamate insecticides ^24– 26^. The spread of this species in Africa despite the widespread implementation of IRS and especially ITNs suggests that there may be a need to look for complementary interventions, particularly given reports of resistance in invasive African populations ^27, 28^. This paper presents an analysis of the literature on vector control interventions against *An. stephensi*. It aims to provide scientific evidence of the efficacy of these interventions with a view to developing an evidence-based integrated control programme for *An. stephensi* in its recently invaded range.

## Methods

### Literature Search methods

This systematic review was conducted following the Preferred Reporting Items for Systematic Reviews and Meta-Analyses (PRISMA) 2020 guidelines ^29^. We performed a systematic search of published literature with no-language restrictions from inception (1976) up to the 5 ^th^ April 2024 using specific search terms. Papers identified were screened and full text versions of relevant studies were obtained. More detail on the search terms isprovided in extended data- supplementary file 1 https://doi.org/10.6084/m9.figshare.27926556.v1 ^30^. A protocol was developed for the systematic search and to establish study selection criteria, but it was not registered.

## Inclusion and exclusion criteria for this review

### Types of studies

We included randomised and non-randomised, controlled epidemiological and entomological studies conducted in communities with wild *An. stephensi* mosquitoes with the following designs:

- Randomised and controlled studies:
  - Individual or cluster randomised controlled trials
  - Step wedge
  - Cross-over design
  - Factorial design
- Non-randomised controlled studies
  - Controlled before-and-after studies
  - Cohort study
  - Case control study
  - Cross-sectional study
  - Time-series or interrupted time-series

We excluded studies conducted in the laboratory as well as studies using laboratory colonies of *An. stephensi*.

### Types of participants

Populations living in rural and urban settings and refugee camps where *An. stephensi* has been reported as an endemic malaria vector or invasive species were considered. Studies involving both adults and children were considered with no restrictions based on age or gender.

### Types of intervention

***Intervention***

We included studies that evaluated ITNs, other insecticide treated materials (e.g. blankets, curtains, wall linings, tents etc), IRS, topical repellents, larvicides, habitat modification, habitat manipulation, biological controls (using predators, pathogenic nematodes) and space spraying. We also included studies that evaluated novel tools; attractive targeted sugar-baits, endectocides, spatial repellents, lethal ovitraps, housing modifications (e.g. untreated or insecticide-treated screening, eave tubes etc) and autodissemination. Interventions based on plant extracts were not considered as dosages have not been standardized and may not be scalable at the present time.

***Control***

Control groups either received no intervention or standard practice vector control interventions (e.g. Insecticide-treated Nets (ITNs) where ITNs are considered standard practice).

### Types of outcome measures

***Primary outcomes***

-Clinical malaria incidence, defined as demonstration of malaria parasites (any *Plasmodium* species) by blood smear or a rapid diagnostic test (RDT), or both; and clinical symptoms including fever or history of fever, detected passively or actively.

-Malaria parasite prevalence, defined as the proportion of surveyed people with *Plasmodium* parasitaemia confirmed by blood smear, RDT, or PCR.

***Secondary outcomes***

### Epidemiological

The occurrence of severe malaria, characterized by at least one of the following: severe anemia (packed cell volume <15%), cerebral malaria (deep coma with a Blantyre coma score ≤2), prostration (inability to sit unaided, seek the mother's breast, or feed in non-sitting children), hypoglycemia (blood glucose <2.2 mmol/L), repeated convulsions (≥2 episodes within 24 hours before admission), respiratory distress (deep breathing or chest indrawing), or hyperparasitemia ( *P. falciparum* infecting >10% of erythrocytes).

Malaria-related hospitalisations: this metric quantifies severe cases requiring inpatient care indicating the overall disease burden.

Malaria related deaths: captures both direct and indirect mortality.

Mean haemoglobin levels (g/dL): represents the severity of anaemia in malaria patients. Lower values indicate more severe infections.

### Entomological

Adult mosquito density is measured using a technique shown to be appropriate for the vector (e.g. human landing catch, CDC light trap, Prokopack aspirator). Adult mosquito density is reported as bites per person per night for human landing catches and mosquitoes per trap per night for trap catches collected during the study period. It refers to the total number of resting mosquitoes collected during the study period using Prokopack aspirators.

Human blood index (HBI) indicates the proportion of blood fed mosquitoes fed on humans out of the total number of mosquitoes fed.

Sporozoite rate is measured as the proportion of vector mosquitoes with *Plasmodium* circum-sporozoite protein (Csp) in their salivary glands. The circum-sporozoite protein can be detected through the enzyme-linked immunosorbent assay (ELISA) method. 

Entomological inoculation rate (EIR) is the estimated number of bites by infectious mosquitoes per person per unit time. EIR is measured as the product of the mean density of mosquitoes obtained by a collection method and the proportion of infected mosquitoes.

Larval density is the number of larvae present in a breeding habitat or a given volume such as per unit of water. Larval density is counted per dip of a water body.

Inhibition of emergence (IE) rate measures the reduction in the proportion of larvae that successfully complete their development and emerged as adults. This variable is determined by the ration of the number of larvae that fail to emerge with the total number of larvae or pupae present multiply by 100.

### Data Extraction and Data management

A data extraction form was used to collect relevant information from the included studies (extended data- supplementary file 2 https://doi.org/10.6084/m9.figshare.27926556.v1 ^30^. Data extraction included study information (e.g. author, publication year, journal, volume, title, region, country, city, study area), trial information (e,g. number of arms, trial design, type of area), outcome of interest, vector species, intervention description (type of intervention, description, dosage, frequency of application) and any other information assessing the impact of intervention (e.g duration of effectiveness, protection time). A narrative and qualitative synthesis were carried out from the selected studies. A narrative synthesis of the findings was performed and structured according to the scope of the review whereas quantitative synthesis was conducted using data tables and graphs. 

To adjust data presentation, (i) available data were used, and missing data were calculated where. For instance, population net coverage was assessed as follows: % population with ITN access = number of ITNs * (1.8/target population) *100. This formula estimates the percentage of the population with access to insecticide-treated nets (ITNs) assuming each net covers approximatively 1.8 people ^31^. (ii) When multiple values of malaria densities or malaria prevalence were provided for various districts within a study area, the average value was calculated and used for analysis. Study quality was assessed using a previously developed tool to analyze the risk of bias categorizing it as either low or high and identifying the type of bias such as selection or performance bias ^32^ (extended data-supplementary file 3 https://doi.org/10.6084/m9.figshare.27926556.v1 ^30^.

## Results

### Scope of the literature

**Figure 1.**
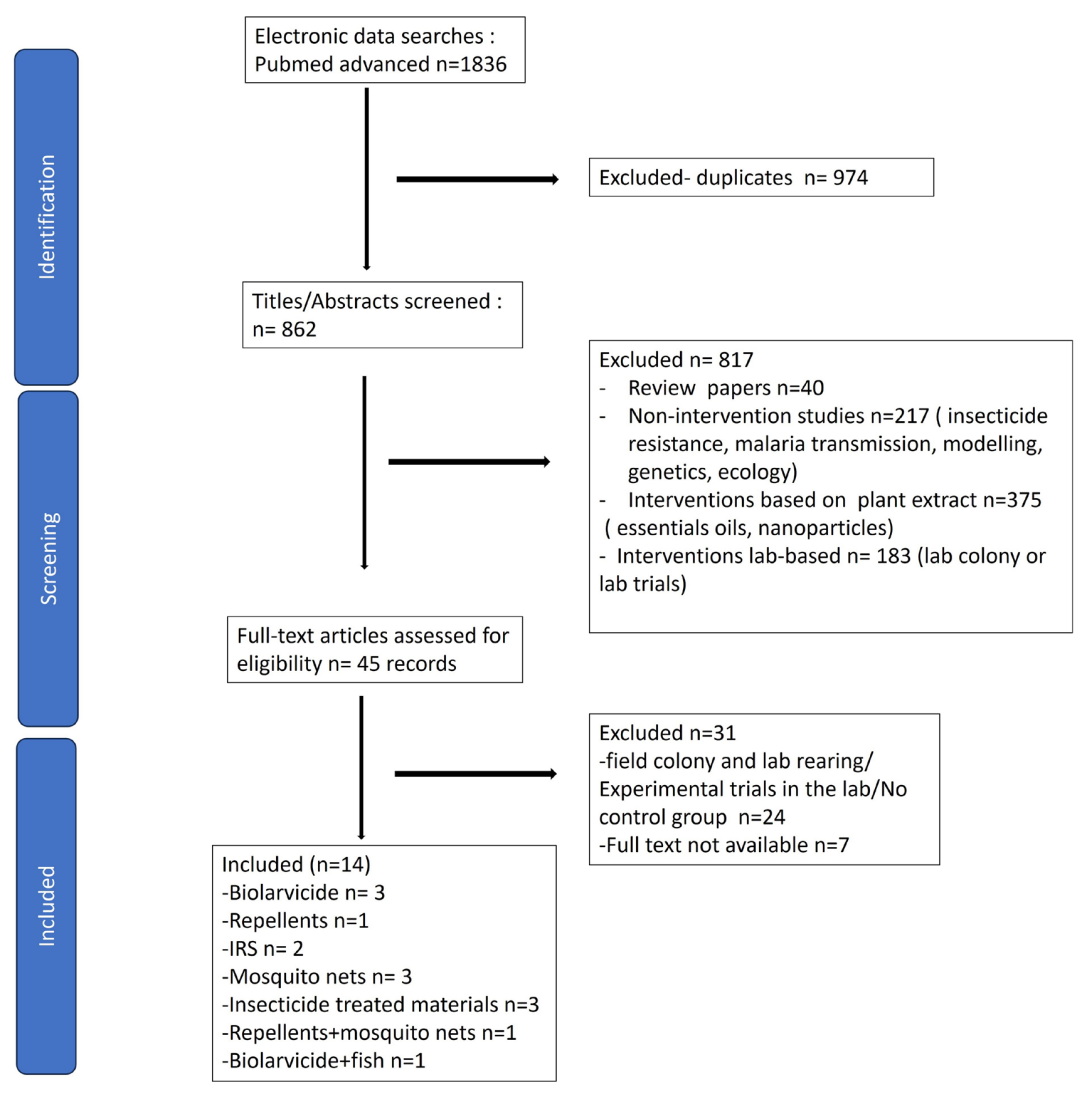
Identification of trials of control measures against *An. stephensi* - PRISMA flowchart of study inclusion.

The systematic search identified a total of 1,836 records ( Figure 1). After eliminating 974 duplicates, we screened 862 records based on title and abstract. Following screening of paper titles and abstracts a further 817 were excluded due to ineligibility/out of review scope. The full text articles were accessed for the remaining 45 records and assessed for eligibility. From the 45 records, 14 full text articles reporting the impact of interventions on *An. stephensi* were analysed ( Figure 1). Table 1 shows the sources by publication year, vector control interventions as well as outcomes measured. Findings reported research from India (n=8), Iran (n=1), Pakistan (n=2) and Afghanistan (n=3).

**Table 1.** Summary of studies presenting efficacy of interventions against *Anopheles stephensi*.

Ref	Country	Year	Study area	Type of area	Intervention	Study design	Outcome measure	Impact on malaria incidence/ prevalence driven by *An. stephensi*	Impact on *An. stephensi* density	*An. stephensi* mortality or other indicators
39	India	1995	Goa	rural	Biolarvicide (Bti)	Before and after control trials	Larval density	NA	Y	NA
40	India	1998	Goa	rural	Biolarvicide (Bti)& Fish (Aplocheilus blocki)	Pre-post treatment	Larval density	NA	Y	NA
38	India	2001	New Dehli	urban	Insecticide treated materials	Pre-post impregnation	Adult densities/ malaria incidence/ prevalence	Y	Y	NA
41	India	2005	Dehli	urban	Insect Growth Regulator (Himilin semiliquid and wettable powder formulations)	NA	Inhibition adult emergence	NA	Y	NA
33	India	2007	Nawada, Durgawali, Harampur	rural	ITNs (PermaNet 2.0)	Before and after trial, controlled	Person-hour adult densities	NA	Y	Y
42	India	2011	Pacheria	rural	repellent (advanced odomos cream & DEET)	Randomised, blinded, control	Human biting rate	NA	NA	Y
43	India	2015	Balepura	rural	Indoor Residual spraying (differents formulations of alpha-cypermethrin)	small scale-controlled trial	Knockdown, Mortality rate, duration of effectiveness	NA	NA	Y
44	India	2018	North & East Bengaluru	urban	Biolarvicide (Bactivec)	Pre-post controlled trial	Larvae and pupae densities	NA	Y	NA
34	Iran	2012	Tisur, Daranar	rural	ITNs (Olyset)	Randomised, controlled, before/After	Mortality rate	Y	Y	NA
36	Afghanistan	2002b	Kabul	urban	Insecticide treated materials	controlled	Blood fed rate/insecticide-induced mortality	NA	NA	Y
35	Afghanistan	2002	Behsud, Chaprahar	rural	ITNs (permethrin or lambacyhalo-thrin)	Cross-sectional/ case-control	Adult densities/malaria prevalence/malaria cases	Y	Y	NA
45	Afghanistan	2004	Dobella	rural	Repellent (DEET) & ITNs	Case-control	Mosquito landing rate/malaria infection	Y	NA	NA
46	Pakistan	2000	Sheikhu-pura	rural	Indoor Residual spraying (alphacy- permethrin wettable powder and suspension concentrate formulations)	Community-randomized controlled trial	Adult mosquito densities/	Y	Y	NA
37	Pakistan	2002a	Peshawar	Urban	Insecticide treated materials	Controlled	Blood fed rate/insecticide-induced mortality	NA	NA	Y

Ref: Reference, Year: publication year , Y: yes, NA: data non-reported

### Interventions reducing human vector contact and associated malaria cases

***Insecticide Treated Nets (ITNs)***

Three studies assessing the efficacy of ITNs (all ITNs) against *An. stephensi* were identified ^33– 35^. These studies reported epidemiological (human blood index, malaria incidence) and entomological data (density of malaria vectors) ( Table 2).

**Table 2.** Effect of insecticide treated materials on malaria cases and *An. stephensi* densities.

						Control areas		Intervention areas	
Areas	Reference	Outcome Variable	Type of study	Type of intervention	Surveillance method	Pre	Post	Pre	Post
Iran	34	Malaria incidence	Cluster randomised	Olyset LLIN	Blood slides examination	104.9	36.9	74.7	2.5
		Malaria cases				99	36	115	4
		Indoor relative abundance (%)			Hand catch and spray sheet collection		136(10.33) ^a^		62(7.76) ^a^
		Outdoor relative abundance (%)			Pit trap and Night biting catch		178(13.52) ^a^		132(16.53) ^a^
		Human blood index (%)			Hand catches		13(28.26) ^a^		6(17.64) ^a^
India	33	Person-hour densities	Before-After trials	PermaNet 2.0 LLIN	Hand-catch	42; 24 ^b^	15; 18 ^b^	61	5
Afghanistan	35	*P. falciparum* prevalence	Cross-sectional surveys	LLIN-treated with permethrin or lambacyhalo-thrin	Blood smear	3.8(68/1811)	4.7(78/2527)	1.9 (25/1143)	2.2(25/1313)
		*P. vivax* prevalence	Cross-sectional surveys		Blood smear	3.4(61/1811)	3.2(78/2527)	4.4 (52/1143)	2.6(21/1313)
		*An. stephensi density per room*	Cross-sectional surveys		Space-sprayed pyrethroid aerosol	1.2(0.9-1.5)	1.4(1.0-1.8)	0.8(0.7-1.0)	0.6(0.5-0.7)
		*P. falciparum* related malaria cases (%)	Case-control Passive surveillance		Microscopy		4.1 ^a^		1.2 ^a^
		*P. vivax malaria* related malaria cases (%)	Case-control Passive surveillance		Microscopy		17 ^a^		14 ^a^
Pakistan	37	Induced reduction in blood feeding (control vs deltamethrin)	Control- treated	Deltamethrin-treated plastic tarpaulin			19.7(5.9-26.4) ^a^		10.1(2.9-13.3) ^a^
India	38	*An. stephensi* densities	Before-After trial	Deltamethrin-treated curtains	Aerosol spray catches	93	39.5	96	7.5

**^a^Variable estimates during the intervention in control and treated areas, ^b^mean densities in untreated and no-nets villages**

Soleimani-Ahmadi *et al.* ^34^ reported a reduction of 93.2% in malaria incidence in the permethrin (Olyset) net areas compared to untreated net areas. The coverage of mosquito nets in each area was 81.6% and 85.7% respectively. Before the intervention, malaria incidence was not significantly different in the two study areas. During the intervention period, malaria incidence drastically decreased in the Olyset area from 74.7 to 2.52 ( Table 2). The human blood index of *An. stephensi* was significantly lower in the Olyset net area compared to the untreated net area (χ ^2^ = 4.57, df= 6, P=0.004) ( Table 2). The intervention was also associated with a significant reduction of 54.4% in the indoor resting density of *An. stephensi.* The same entomological trend was observed in India following the distribution of PermaNet ITNs ^33^. The mean person-hour densities of *An. stephensi* significantly decreased during the post-distribution period (P<0.0001) in PermaNet villages from 61 mosquitoes per hour per month (mos/h/m) to 5 (mos/h/m) compared to the control groups.

In another study conducted in Afghanistan ^15^ where the net coverage estimates were 57% and 34% during the cross-sectional survey and passive surveillance respectively, authors reported a significant reduction of the prevalence of *P. vivax* from 4.4% to 2.6% among insecticide treated net (ITN) users while it remained stable among non-users ( Table 2). The individual protective effectiveness of ITN against *P. falciparum* were 59% and 69% during the cross-sectional surveys and the passive surveillance case-control respectively and against *Plasmodium vivax* 50% and 25% (P<0.05 in both cases).

***Others insecticide treated materials***

Insecticide-treated materials such as sheets, blankets and curtains were assessed as protective tools against *An. stephensi* in refugee camps in Pakistan and Afghanistan ^36, 37^ as well as in an urban community of India ^38^.

Graham *et al.* ^37^ compared three cotton blankets impregnated with different pyrethroids (permethrin, deltamethrin and alphacypermethrin) for efficacy against *An. stephensi* and other species in a refugee camp in Pakistan. The proportion of blood-fed mosquitoes was lower at the treatment sites compared to the untreated sheets but only significant with deltamethrin (48.7% induced reduction, P<0.05) ( Table 2). The mean mortality rate of *An. stephensi* was 44.4% on treated blankets with a treatment-induced mortality of 28.3% which was higher than the 22.4% mortality rate recorded on untreated blankets (P<0.05). The same trend was observed in another study conducted in Afghan refugee camps ^36^ with high mortality rates of *An. stephensi* in sites with pre-treated sheets (mean mortality- 94%) compared to control (5%). The mean blood-feeding rate did not differ between the treated sheet and control arms (P=0.82).

A before-and-after field trial study in the New Delhi municipality evaluated the effect of a deltamethrin-treated curtain at 100 mg/m ^2^ on *An. stephensi* densities and associated malaria cases ^38^. Malaria incidence in deltamethrin treated localities was reduced by 93% and 98.7% following the first and second impregnation, respectively giving an overall reduction of 95.4%. A significant reduction in *An. stephensi* indoor resting density was recorded (P<0.05) ( Table 2) with a 96.9% reduction during the first impregnation and 82% during the second, resulting in an overall reduction of 93.1% ^38^. The authors also reported that the protective effectiveness of deltamethrin-impregnated curtains against *An. stephensi* is 3 months after the first impregnation and 5 months after the second one. An accompanying community survey following the deployment of the deltamethrin-treated curtains showed high acceptability of the strategy (data not shown) ^38^.

### Efficacy in reducing *An. stephensi* densities

***Indoor Residual spraying (IRS)***

IRS has been used to control *An. stephensi* in Pakistan, India and Iran ^43, 46, 47^. A pre and post community controlled randomised IRS trial was implemented in Pakistan in June 1997 ^46^ using alpha-cypermethrin wettable powder (WP) and suspension concentrate (SC) formulations at 25 mg AI/m2. During the pre-intervention period, there was no significant difference between the treatment and the controls groups (P=0.81 and P=0.29 for *P. falciparum* and *P. vivax* malaria). After the intervention, the incidence of *P. falciparum* malaria remained below 3 per 1000 person years (ppy) in the treatment groups while rising to 29 ppy in the control groups (P=0.02) ( Table 3). The same trend was recorded with incidence of *P. vivax* malaria. In this trial, the protective efficacy was 90–95% against *P. falciparum* malaria for SC and WP insecticide formulations respectively and around 80% against *P. vivax* malaria for both. The authors also reported a reduction in *An. stephensi* mean densities of 51% and 68% for the SC and WP formulations respectively with an observed residual efficacy of 4 months against *An. stephensi*. 

**Table 3.** Impact of IRS on clinical outcomes and *An. stephensi* densities.

				**Control**		**SC**		**WP**		**PE**
		Type of study	Surveillance methods	Pre	post	pre	post	pre	post	
46	*P. falciparum* malaria Incidence	Community randomised Controlled Before-after trials	Blood smear	5.4	29.5	5.3	2.7	2.0	1.4	90–95%
	*P. vivax* malaria incidence		Blood smear	56	18.7	70	4.2	44	3.7	80%
	*P. falciparum* prevalence		Blood smear	0.7	3.9	1.1	0.0	0.5	0.6	
	*P. vivax* prevalence		Blood smear	6.4	7.5	5.3	2.0	3.7	2.7	
	Mean densities [95% CI]	Community randomised controlled trials	PSC-based density assessment	29 [24,35]		14 [5,33]		9 [4,19]		

PE: Protective Efficacy, 100*(1-IRR)%; WP: Wettable Powder; SC: Suspension Concentrate

A small-scale (Phase II) field trial in India ^43^ suggested that alpha-cypermethrin WG-SB, a water-dispersible granular formulation packed in water-soluble bags provided 13–16 weeks of residual efficacy against *An. stephensi* while the WP formulation provided 11–15 weeks on most common indoor surfaces.

***Repellents***

Two studies reported the evaluation of repellents against *An. stephensi,* one in India and one in Afghanistan ^45, 48^. In India, Mittal *et al.* ^48^ assessed the efficacy of DEET (12% Diethyl-3-methylbenzamide) and Odomos Cream (12% N.N-diethyl-benzamine) against *An. stephensi* in a randomised, blinded, field-controlled trial. Following random selection of houses and volunteers, volunteers and mosquito collectors were blinded to doses and repellent creams. After testing different concentrations of cream from 1 to 12 mg/cm ^2^, they found a 100% protection up to 11h at 10 mg/cm ^2 ^against *An. stephensi* with no significant difference between the two creams (P>0.05).

A different formulation called Mosbar containing 20% DEET and 0.5% permethrin was tested in Afghanistan during a case-control study ^45^. The authors assessed the protective effect of Mosbar and/or insecticide treated Nets (ITNs). They reported a 20.2% rate of Mosbar use among the control group greater than the 11.5% in cases group indicating the uptake of Mosbar in the study area where ITN coverage was 66 %. Their findings highlighted that using Mosbar or ITN led to significant reductions of 50% (P<0.001) and 48% (P=0.003), respectively. In comparison, the combined use of Mosbar and ITN resulted in a 69% reduction in the odds of malaria (95% CI: 28% to 87%) after adjusting for other unadjusted factors. However, the additional benefit of using both Mosbar and ITN together compared to using ITN alone (P=0.68) or Mosbar alone (P=0.18) was not statistically significant. protective efficacy was 31% for the combined intervention, 50% for Mosbar and 52% for ITNs.

***Biological larvicide***

Three studies assessed the efficacy of the biological larvicide *Bacillus thuringiensis* (Bti) ^39, 44^ or Bti in combination with the larvivorous fish *Aplocheilus blocki* ^40^. Field trials of Bactivec® SC (M/s Labiofam Entreprise Group, La Habana) were conducted in India at a dose of 1ml/50l using a hand atomiser sprayer or graduated pipette, depending on the size of breeding sites. The test formulation Bactivec SC contains *Bti* serotype H-14, strain 266/2 as active ingredient (6 g/l insecticidal toxins and spores; and 994 g/l other ingredients). Field application of that biolarvicide was associated with an 80–96% reduction in larval density and 81–100% reduction in pupal density in study areas ^44^. The authors also reported residual activity of 7 to 14 days against *An. stephensi* with a lower dosage of 0.5ml/50 l and 14–17 days with 1ml/50l during a 24 day follow on large-scale trial. No significant difference was observed between the two dosages in reducing the density of larvae and pupae across the two habitat types tested, indicating that both dosages were equally effective in controlling immature stages ^43^. Kumar *et al.* ^39^ tested the effect of *Bti* at a dosage of 1g/m ^2^ on *An. stephensi* in Goa, India. Within 24 hours of application, 97.8% mortality of third and fourth instar larvae was observed in treated areas. Low densities were observed until day 35 after treatment. No pupae were observed in the treated habitats for up to 21 days until the end of the study ^39^. The same authors observed a significant reduction of 396 malaria cases (χ ^2^ = 712, P < 0.001) following the introduction of fish *Aplocheilus blocki* and weekly spraying of *Bti*, when comparing malaria incidence from the pre-treatment period to the treatment period ^40^. Malaria slide positivity rates (SPR) also declined by 6.83% (χ ^2^ = 10.36, P < 0.001) during the post-treatment period. Overall, by comparing malaria incidence in the experimental areas with nearby endemic towns, authors reported that the slide positivity rate, slide *Plasmodium* positivity rate and parasite index reduction rate were 57.3%, 82.6% and 81.6% respectively after implementation of the two interventions.

Introducing fish at a dosage of 5 fish/m ^2^ into naturals habitats reduced larval density from 16.2 per dip during the pretreatment period to 0.65 per dip (t=2.9, P=0.002) corresponding to a decline of 0.96 % of larval density ^40^.

***Insect growth Regulators***

Ansari *et al.* evaluated the efficacy of an insect growth regulator, Hilmilin (diflubenzuron) against *An. stephensi* in India ^41^. Two doses of 0.04 and 0.08 g/m ^2^ were sprayed weekly in breeding habitats to assess the inhibition of adult emergence. A 100% inhibition of adult emergence was achieved against *An. stephensi* for up to 6 weeks ^41^.

## Discussion

This review summarises data from all published studies of interventions against *An. stephensi* that we were able to identify using a standardised search methodology ^29^.

All data retrieved from the literature reported research from the native range of *An. stephensi* and were published between 1995 and 2018. According to our search, no studies have been performed to date in Africa where *An. stephensi* has expanded since 2012 ^7, 49^ and malaria cases have been associated ^21, 50^. This indicates a major knowledge gap in terms of entomological and epidemiological data that can inform interventions and policy guidelines for controlling the invasive Asian vector in Africa.

### Effectiveness in reducing malaria cases or prevalence

Clinical evidence on the effectiveness of control interventions in reducing malaria cases or malaria incidence has been assessed in case-control studies in Afghanistan ^35, 45^ and community randomized controlled trials in Pakistan ^46^. These interventions tested ITNs and repellents using Mosbar. Community use of Mosbar reduced the likelihood of *P. falciparum* malaria, with a protective efficacy of 56% the effect against *P. vivax* malaria was not significant (protective efficacy of 29%) ^45^.

A significant impact of ITNs against malaria prevalence and incidence was highlighted in the literature ^15, 34^. Despite varying levels of ITN coverage in these studies, a significant impact was observed. In Afghanistan, malaria prevalence was significantly lower among individuals who used ITNs ^15^. Similarly, results from a study in Iran showed a substantial reduction in both indoor and outdoor densities of *An. stephensi* densities in areas with Olyset nets compared to those with untreated nets ^34^. These findings support the effectiveness of ITNs in areas with adequate LLIN coverage ^51^. This clearly demonstrates that high ITN coverage substantially reduces malaria transmission providing a community-wide effect by reducing the number of infective mosquitoes ^52^. Using a previously published risk of bias assessment form ^32^, our analysis showed a low risk of bias in these trials.

### Interventions that reduce human-vector contact

Several interventions have been reported to prevent malaria by reducing human-vector contact and malaria transmission in human populations. In addition to ITNs, some studies have shown a beneficial effect of repellents and insecticide-treated materials against *An. stephensi* mosquitoes and others malaria vectors. *Anopheles stephensi* co-occurs with a number of other malaria vectors such as *An. culicifacies*, *An. dthali*, *An. nigerimus*, *An. subpictus* ^34, 36, 37^ across Southeast Asia, Iran and Pakistan ^53^.

Data showed complete protection against *An. culicifacies and An. stephensi* for up to 11 hours following the application of Advanced Odomos and DEET cream ^48^. Evidence for the efficacy of insecticide-treated materials against *An. stephensi* and local vectors has predominantly been reported in refugee camps in Pakistan and Afghanistan where people are more likely to sleep in exposed situations ^36, 37^. These individuals may have limited access to health services and supplies, and the tents provided for their shelter may offer minimal protection from mosquitoes ^54^. Housing improvements and protective clothing can also reduce human-vector contact and control malaria as previously reported ^55, 56^. However, no studies were identified in this review using these measures to prevent exposure to *An. stephensi* bites or associated malaria cases.

In addition to the impact of insecticide-treated materials in protecting against *An. stephensi*, additional effects were observed against *Aedes aegypti* in India. Deltamethrin-treated curtains significantly reduced the indoor resting density of *Aedes aegypti* by 93.7% ^38^. Similarly, Mittal *et al.* ^48^ reported that advanced Odomos and DEET provided complete protection against *Aedes aegypti* for up to 6 hours.

### Effectiveness in reducing *An. stephensi* densities

Literature-based evidence supports the effectiveness of different control tools in reducing *An. stephensi* densities at both immature and adult stages ^43, 46, 47^. These interventions included long-lasting insecticide treated nets (ITNs), indoor residual spraying (IRS) and biological control using insect growth regulators (IGR). The use of ITNs progressively reduced adult *An. stephensi* densities to 54.4% in a pre-post intervention trial in India ^33^, and 68% in another community randomised controlled trial in Pakistan ^46^. In India, reductions in larval and pupal densities have been reported in some settings within 24h of treatment using a biolarvicide, *Bacillus thurigiensis* ^44^.

### Integrated control measures

Integrating different interventions can have synergistic effects, that may enhance overall cost-effectiveness. In Afghanistan, the combined use of a DEET mosquito repellent and bed nets resulted in a 69% [95% CI: 28-87%] reduction in the likelihood of malaria, whereas the use of either mosquito repellent or bed nets alone resulted in reductions of 50% and 48%, respectively ^45^. However, the added benefit of using both DEET and ITNs together compared to using either ITNs or DEET alone was not statistically significant. Another study in India combining the use of a biolarvicide, *Bacillus thurigiensis*, and a larvivorous fish also reported a significant impact on *An. stephensi* populations and subsequent malaria transmission ^40^. The cost-effectiveness of integrated approaches requires further evaluation, as it is influenced by local vector ecology, insecticide resistance trends, and the practicality of implementation with simultaneous management of different interventions potentially increasing operational complexity.

Although our database is comprehensive, our review has some limitations. We focused on the combination of *An. stephensi* and existing interventions as keywords and only included published papers. We may have missed some papers that covered the topic of interest but didn’t include *An. stephensi* as a keyword or in the title. We faithfully reported the data from the original publications without any additional analysis (adjustment of P value or protective efficacy). The indicators differed between studies, and the study designs were not always comparable. In some studies, a mean density was calculated if there were multiple values before or during the intervention.

## Conclusions

The literature provides strong evidence that Insecticide Treated Nets (ITNs) and Indoor Residual Spraying (IRS) are effective in controlling malaria and *An. stephensi* in its native range, whilst repellents show promise as a complementary control measure. The private sector could play a critical role in scaling up the production and distribution of repellents in Africa, which experiences the spread of invasive species and high incidence of vector borne diseases, offering an affordable, widely accessible option for malaria prevention. Addressing the gap of cost-effectiveness analysis is also crucial for optimizing resources and improving the overall impact of malaria vector control efforts. In addition, there is a need for additional epidemiological evidence to support deployment of interventions against *An. stephensi*, especially in African settings.

## Ethics and consent

Ethical approval and consent were not required.

## Data Availability

All data are available as part of the article. Figshare: Extended data for “ A systematic review of interventions targeting Anopheles stephensi. https://doi.org/10.6084/m9.figshare.27926556.v1 ^30^ This dataset contains the following extended data: Flowshart.PNG **Supplementary file 1:** Search terms (XLSX) **Supplementary file 2:** Data extraction form (DOCX) **Supplementary file 3:** Risk of bias assessment form (DOCX) **Supplementary file 4:** Details of results presented in Table 1 (XLSX) Figshare : Supplementary file 5- PRISMA checklist for “A systematic review of interventions targeting Anopheles stephensi” https://doi.org/10.6084/m9.figshare.27926556.v1 ^30^ Data are available under the terms of the Creative Commons Zero "No rights reserved" data waiver (CC0 1.0 Public domain dedication).
