# Supplementary material for: A systematic review of interventions targeting Anopheles stephensi
Source: Wellcome Open Res. 2024 Dec 16;9:724. [Version 1] doi: 10.12688/wellcomeopenres.23480.1 (PMC11751610; doi:10.12688/wellcomeopenres.23480.1)
Supplement: Peer review history

# Reviewer report: Reviewer response for version 1

**DOI:** 10.21956/wellcomeopenres.25894.r115869

**Published:** 2025-01-21

**Authors:** Basil D. Brooke

**Affiliations:** 1 Centre for Emerging Zoonotic and Parasitic Diseases, National Institute for Communicable Diseases (NICD), National Health Laboratory Service (NHLS), Johannesburg, South Africa

I believe this manuscript provides useful and important information on the control and localized elimination of *An. stephensi*, a malaria vector that has increased its range from South Asia and the Middle East into various parts of Africa. I do however think that the authors should acknowledge in the Abstract conclusion that IRS and ITNs have thus far only proved effective against *An. stephensi* in South Asia and the Middle East, and that the efficacies of these interventions have not yet been demonstrated in any African settings.   

 I have only minor concerns for the authors to address as listed below:

**Page 5: **Methods/secondary outcomes/Entomological/6 ^th^ paragraph – change ‘ration’ to ‘ratio’ and ‘multiply’ to ‘multiplied’

**Page 5:** Methods/secondary outcomes/data extraction and data management/2 ^nd^ paragraph – Fix punctuation in first sentence

**Page 9: **Repellents/2 ^nd^ paragraph – the 3 ^rd^ sentence is unclear. What were the control and cases groups? In the 4 ^th^ sentence, ‘reductions’ of what? malaria incidence?

**Page 10:** Effectiveness of reducing malaria cases or prevalence/1 ^st^ paragraph/last sentence – delete ‘the effect’

**Page 10:** Effectiveness of reducing malaria cases or prevalence/2nd paragraph/4th sentence – delete 2 ^nd^ use of the word ‘densities’

Are the rationale for, and objectives of, the Systematic Review clearly stated?

Yes

Is the statistical analysis and its interpretation appropriate?

Not applicable

If this is a Living Systematic Review, is the ‘living’ method appropriate and is the search schedule clearly defined and justified? (‘Living Systematic Review’ or a variation of this term should be included in the title.)

Not applicable

Are sufficient details of the methods and analysis provided to allow replication by others?

Yes

Are the conclusions drawn adequately supported by the results presented in the review?

Yes

Reviewer Expertise:

Vector biology, ecology and control/elimination, especially malaria vectors

I confirm that I have read this submission and believe that I have an appropriate level of expertise to confirm that it is of an acceptable scientific standard.

# Reviewer report: Reviewer response for version 1

**DOI:** 10.21956/wellcomeopenres.25894.r116417

**Published:** 2025-01-20

**Authors:** Oscar Mbare

**Affiliations:** 1 International Centre of Insect Physiology and Ecology, Nairobi, Kenya

I enjoyed reading this paper. The article is well written and easy to read and follow. It is an interesting paper that provides evidence on the control measures targeted at *An. stephensi*, the invasive *Anopheles* species in Africa.   

 I have the few comments below for the authors:

1. Please include additional information in the results section of the abstract to summarize the findings of the studies reviewed.
2. In the conclusion section there is no mention of larval control approaches that were reviewed. The reviewed studies focused on larval control measures reported that this strategy also reduced the density of malaria vectors and malaria cases. It would be nice to also have this captured in the abstract as well as conclusion in the main text. 
3. Table 3: Please add the word ‘vector or *Anopheles’* in the outcome measure in the last column named ‘Mean densities.

Are the rationale for, and objectives of, the Systematic Review clearly stated?

Yes

Is the statistical analysis and its interpretation appropriate?

Yes

If this is a Living Systematic Review, is the ‘living’ method appropriate and is the search schedule clearly defined and justified? (‘Living Systematic Review’ or a variation of this term should be included in the title.)

Not applicable

Are sufficient details of the methods and analysis provided to allow replication by others?

Yes

Are the conclusions drawn adequately supported by the results presented in the review?

Yes

Reviewer Expertise:

Medical entomology

I confirm that I have read this submission and believe that I have an appropriate level of expertise to confirm that it is of an acceptable scientific standard.
